# Understanding the Impact of Screening Location on Depressive Symptom Scores Among People With Spinal Cord Injury

**DOI:** 10.1016/j.arrct.2026.100592

**Published:** 2026-01-30

**Authors:** Ramya Gopalan, Elizabeth Pasipanodya, Benjamin Dirlikov, Mark Held, Janelle Myhre, Cria-May Khong, Kazuko Shem

**Affiliations:** a Rehabilitation Research Center, Santa Clara Valley Medical Center, San Jose, CA.; b Department of Psychiatry, Santa Clara Valley Medical Center, San Jose, CA.; c Department of Physical Medicine and Rehabilitation, Santa Clara Valley Medical Center, San Jose, CA.

**Keywords:** Community reintegration, Depression, Nontraumatic spinal cord injury, Rehabilitation, Suicidal ideation, Traumatic spinal cord injury

## Abstract

**Objective:**

To compare Patient Health Questionnaire-9 (PHQ-9) responses among individuals screened in inpatient rehabilitation versus at home during the first year after spinal cord injury (SCI).

**Design:**

Cross-sectional secondary analysis.

**Setting:**

Depressive symptomology was assessed either during SCI acute inpatient rehabilitation stay (Inpatient Group [IG]) or postdischarge in home setting (Home Group [HG]).

**Participants:**

A total of 148 individuals (N=148) with traumatic or nontraumatic SCI with complete PHQ-9 responses (42 HG; 106 IG) were included. Participants were 71% men, with a mean age of 48±19.5 years and a duration of injury (DOI) 62±55 days (between April 2019 and January 2024).

**Interventions:**

Not applicable.

**Main Outcome Measures:**

PHQ-9 scores and prevalence of suicidal ideation (SI).

**Results:**

Median DOI in the IG was 35 days versus 78 days in the HG (*P*<.001). PHQ-9 total scores and the rate of probable major depressive disorder did not differ between the 2 groups (*P*=.09 for both); however, the median subscale score for nonsomatic (affective) items of the PHQ-9 was significantly greater in the HG (3.0) versus IG (1.0; *P*=.004). SI was greater in the HG (21%) versus IG (9%; *P*=.049). Furthermore, among those endorsing SI (N=19), the HG reported a significantly greater severity of SI based on median PHQ-9 (item 9) scores compared with the IG (2.0 vs 1.0; *P*=.035). Post hoc analyses indicated screening location as a significant predictor of nonsomatic subscores (*P*=.008) as well as SI (*P*=.029), after adjusting for DOI.

**Conclusions:**

Elevated nonsomatic depressive symptom scores, including greater SI in the HG, highlight the heightened vulnerability of individuals transitioning from inpatient rehabilitation to home during the first year after SCI. These findings emphasize the need for systematic mental health screening during the early phase of community reintegration.

Spinal cord injury (SCI) is a life-altering event that can pose significant physical, social, and psychological challenges, which can increase the risk of mental health distress, including depression and suicidal ideation (SI).^1^, ^2^, ^3^, ^4^, ^5^, ^6^ Estimates note an increased 22.2-percentage-point prevalence of depression in individuals with SCI, in comparison with an estimated 7% in the general US population.^7^^,^^8^ The rates of depression after SCI have additionally ranged from 9.8% to 63.9% depending on demographic and injury characteristics, sampling methods, study designs, diagnostic tools, and methodologies.^1^^,^^3^^,^^7^^,^^9^, ^10^, ^11^, ^12^, ^13^ Prevalence of SI is estimated at 13.3% among individuals with SCI compared with 3.7% among adults in the general US population.^2^^,^^14^ Yet, similar to the rates of depression, estimates of SI also have widely ranged between 4% and 67% across studies, depending on participant and study-related factors.^15^

The heterogeneity in rates may also be contextual, as in the case of community reintegration, where beyond the physicality of injury, personal, social, and environmental factors can differently impact the complex and continuous process of adaptation.^16^ Although no definitive methods exist to predict how an individual will adapt to the circumstances following SCI,^17^^,^^18^ most adjustment is assumed to occur within the first 2 years of injury.^1^^,^^19^, ^20^, ^21^ However, a critical period of adjustment occurs when newly injured individuals transition from acute inpatient rehabilitation to community residences, where they may encounter stark changes in care and support that may differentially impact mental health.^22^ For instance, the comprehensive support and specialized care during acute rehabilitation offer a buffered and structured environment for patients to adjust to a new SCI.^23^, ^24^, ^25^, ^26^ The contrasting shift postdischarge to a less-resourced system in the community can present psychosocial challenges, exacerbating the risk of depression.^13^^,^^19^^,^^23^^,^^24^^,^^27^^,^^28^

Early identification of patients at risk for depression can support accurate diagnosis and early treatment that can improve mental health outcomes.^29^ Despite marked differences in care and support between hospital and home settings, limited research on depression after SCI has explored differences in the incidence and severity of depression and SI, during and shortly after inpatient rehabilitation.^11^ Extant studies on depression after SCI, as such, have largely been conducted either in inpatient rehabilitation/hospital settings or well beyond the first year of injury in home or community settings.^1^

Therefore, the objective of this cross-sectional study was to compare (1) the profile of depressive symptomatology, based on Patient Health Questionnaire-9 (PHQ-9) scores; (2) prevalence of probable major depressive disorder (PMDD); and (3) prevalence of SI, in individuals with SCI in acute rehabilitation versus those residing in home settings during the first year of injury. We hypothesized that individuals with SCI would exhibit greater depressive symptomatology scores and higher rates of PMDD and SI postdischarge, compared with during acute inpatient rehabilitation.

## Methods

### Participants and procedures

Participants in this secondary analysis were individuals who were screened for eligibility in a clinical trial (NCT 03711760) examining the efficacy of telepsychology cognitive behavioral therapy on depressive symptomatology and well-being.^30^ Most individuals screening for study participation were recruited from an acute SCI rehabilitation unit, at a level 1 trauma hospital, except for 5 individuals recruited via community outreach efforts. All participants provided informed consent, and the study was approved by the local institutional review board.

**Fig 1:**
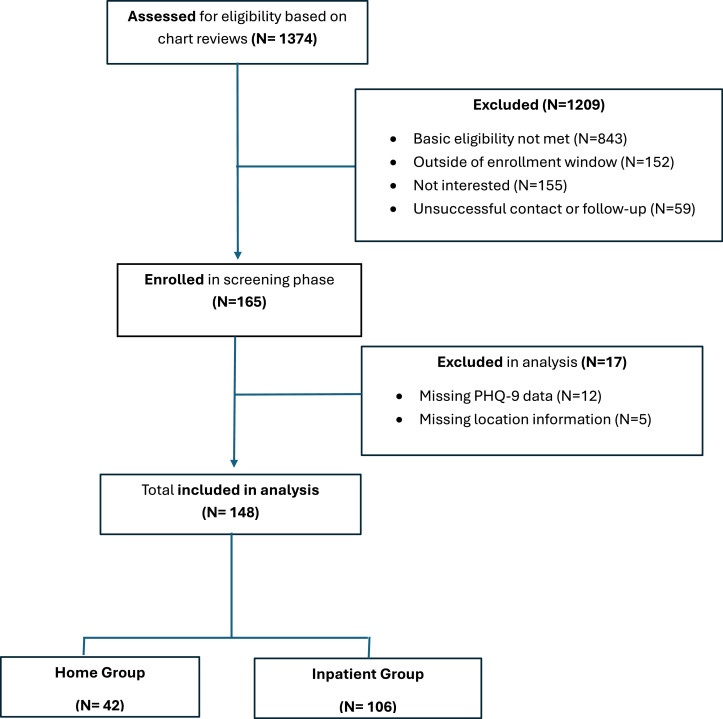
Flow diagram of study participation and final inclusion in the analysis.

Basic eligibility criteria for screening into the clinical trial were (1) age ≥18 years; (2) SCI of any etiology and level; (3) within the first year of injury; (4) discharging to or residing in a private residence in California; and (5) English speaking. Exclusion criteria included medical instability and acute psychosis, based on review of medical records or the treating physician’s judgment. As can be seen in figure 1, 165 eligible individuals signed screening consents, whereas 155 declined participation because of “noninterest,” between April 2019 and January 2024. Screening procedures included administration of the PHQ-9 by research staff, either in-person during acute rehabilitation or postdischarge via telephone or abstracted from participants’ electronic medical record when clinically administered by hospital staff within 2 weeks of study consent. Twelve individuals without PHQ-9 data (not collected because of ineligibility per physician judgment or revocation of interest to participate in PHQ-9 screening after consent) and 5 others with unascertainable screening location were excluded from these analyses. Thus, 148 participants were included in this cross-sectional study, 106 of whom were screened during inpatient rehabilitation stay (Inpatient Group [IG]) and 42 participants were screened at home after discharge (Home Group [HG]). Figure 1 details participant eligibility and inclusion in the final analysis.

### Measures

#### Demographic and injury data

Demographic and injury-related data, including age, sex-at-birth, injury level (tetraplegia or paraplegia), etiology (traumatic or nontraumatic), completeness of SCI (complete or incomplete), duration of injury (DOI), and length of stay (LOS) were abstracted from available medical records. In the HG, 3 participants had missing ages and injury data, whereas LOS was missing for 4 participants. All abstracted data were manually entered into the study database and quality-checked for accuracy by the research team.

#### Depression and SI

The PHQ-9 is a convenient 9-item assessment instrument for screening depressive symptomatology,^31^ deemed to be psychometrically reliable and valid for use among individuals with SCI across multiple settings.^32^, ^33^, ^34^, ^35^ The PHQ-9 scores range from 0 to 27, measuring the severity of depressive symptoms. Each item response was rated from 0 to 3 (not at all, several days a week, more than half the days, and nearly every day) during the last 2 weeks. Scores ≥10 have been used as cut-offs to define PMDD, with good sensitivity and specificity (0.88 for each).^36^ Severity of depressive symptoms, based on total scores, was categorized as minimal (1-4), mild (5-9), moderate (10-14), moderately severe (15-19), and severe (20-27).^37^ SI was measured using item nine of the PHQ-9, with scores of 1 (“several days”) or greater suggesting SI.

**Fig 2:**
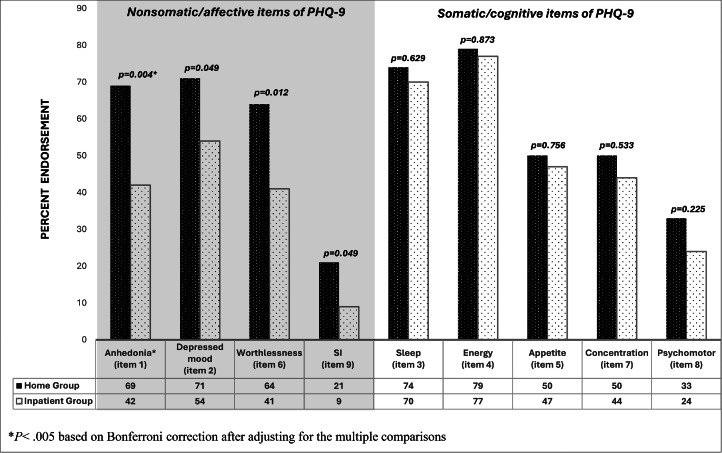
Post hoc exploratory analysis comparing nonsomatic versus somatic PHQ item endorsement patterns between home and inpatient groups. *Shaded plot area represents PHQ-9 non-somatic affective items; Unshaded plot area represents PHQ-9 somatic/cognitive items.*

The somatic symptoms of the PHQ-9 can closely overlap with the secondary complications of SCI.^38^, ^39^, ^40^ Therefore, to understand if the HG and IG can be distinguished based on symptom factors, the nine PHQ-9 items were categorized into 2 subscales: somatic/cognitive (1 cognitive and 4 somatic symptoms) and nonsomatic (4 affective symptoms) based on “factor structure” by Kraus et al,^37^ tested in the community among 7296 SCI individuals. This was the best fit model with the lowest root mean square error of approximation (0.054) among other 2-factor models using all nine PHQ-9 items. Figure 2 describes the items categorized by the 2 subscales.

### Statistical procedures

Descriptive statistics were used to summarize available demographic, injury, and PHQ-9 data for the overall group, HG and IG (Table 1, Table 2). Tests of normality were conducted using the Shapiro-Wilk test to ascertain the use of appropriate statistical methods for comparisons between the HG and IG; no continuous variables met assumptions of normality. Thus, medians, interquartile ranges, and Mann-Whitney *U* tests were used for continuous variables. For categorical variables, percentages are reported, and χ^2^ tests were used to examine differences. A 2-tailed *P* value <.05 was considered for establishing statistical significance for a priori hypotheses.

**Table 1:** Demographic and injury characteristics.

Characteristic	Total (n=148)	IG (n=106)	HG (n=42)	Test Statistic	*P* Value
Age (y)	46.0 (28.3-66.3)	49.5 (27.2-64.2)	46.0 (30.4-68.0)*	*U*=2039.0	.9
Sex-at-birth (male)	70.9% (105)	71.7% (76)	69.0% (29)	χ2=.103	.75
DOI (d)	43 (29-78)	35.0 (24.7-52.2)	78.0 (59-120)*	*U*=746.5	<.001‡
Paraplegia	52.7% (78)	56.6% (60)	42.9% (18)*	χ^2^=1.25	.263
Incomplete level	74.3% (110)	75.5% (80)	71.4% (30)*	χ^2^=.033	.856
Traumatic SCI	58.8% (87)	61.3% (65)	52.4% (22)*	χ^2^=.286	.593
Rehab LOS (d)	27.0 (20-35)	27.0 (21-36)	25.0 (17.5-34.2)†	*U*=1809.5	.354

NOTE. Values are expressed as median (interquartile range) for continuous variables and % (n) for categorical variables.

Abbreviations: *U*, Mann-Whitney *U* test for continuous variables; χ^2^ for categorical variables.

⁎ Missing data on 3 participants for age, DOI, level, completeness, and type of injury in the HG (n=39).

† Missing data on 4 participants for Rehab LOS in the HG (n=38).

‡ *P*<.05 (2-tailed).

**Table 2:** Differences in depression severity and SI between Home and Inpatient groups based on the PHQ-9 scores.

Measure	Total (n=148)	IG (n=106)	HG (n=42)	Test Statistic
PHQ-9 Total Score	6 (3-11)	6 (3-10)	7.5 (4-12)	*U*=1831, *P*=.09
PMDD (PHQ-9≥10)	29% (43)	25.5% (27)	38% (16)	χ^2^(1)=2.33, *P*=.09
Depression symptom severity				χ^2^ (5)=3.72, *P*=.59
None (0)	10% (15)	10% (11)	9% (4)	
Minimal (1-4)	25% (37)	26% (28)	21% (9)	
Mild (5-9)	36% (53)	38% (40)	31% (13)	
Moderate (10-14)	18% (26)	17% (18)	19% (8)	
Moderately severe (15-19)	9% (13)	7% (7)	14% (6)	
Severe (20-27)	3% (4)	2% (2)	5% (2)	
Somatic/cognitive subscale	4 (2-7)	4 (2-6)	4 (2-7.25)	*U*=2125, *P*=.667
Nonsomatic subscale	2 (0-4)	1 (0-3)	3 (1-5.25)	*U*=1558, *P*=.004*
SI				
(PHQ9_Q9>0)	13% (19)	9% (10)	21% (9)	χ^2^ (1)=3.87, *P*=.049*
PHQ9_Q9 score†	1(1-2)	1 (1-1)	2(1-2)	*U*= 24, *P*=.035*

NOTE. Values are expressed as median (interquartile range) or as % (n).

Abbreviations: *U*, Mann-Whitney *U* test, χ^2^.

⁎ 2-tailed *P*≤.05.

† Data on 19 individuals with SI (PHQ-9 Q9>0); n=9 (HG); n=10 (IG).

As part of post hoc exploratory analyses, a series of χ^2^ tests was conducted on individual symptoms of the PHQ-9 items comparing the HG and IG. For these multiple comparisons, an adjusted significance threshold of *P*<.0056 (0.05/9) was applied based on Bonferroni correction. Furthermore, the impact of location (HG vs IG) on SI as well as nonsomatic subscores, after controlling for DOI, was assessed using post hoc linear regression (Table 3, Table 4). Median imputations were used to address missing DOI among 3 individuals for the linear regression analyses. Unstandardized coefficients (b values), standardized beta values, and *P* values were reported. All statistical analyses were done using SPSS version 27.0.^a^

**Table 3:** Post hoc regression analysis of SI based on PHQ-9 (item 9): effect of screening location and DOI.

Model	Unstandardized Coefficients		Standardized Coefficients	*t*	Sig.
	B	Std. Error	β		
(Constant)	.071	.063		1.137	.258
Screening location (HG=1; IG=0)	.219	.099	.196	2.205	.029*
DOI	.001	.001	.072	.813	.418

NOTE. Dependent variable: PHQ-9 item 9 (SI score).

⁎ *P* value significant at <.05.

**Table 4:** Post hoc regression analysis of nonsomatic symptoms: effect of screening location and DOI.

Model	Unstandardized Coefficients		Standardized Coefficients	*t*	Sig.
	B	Std. Error	β		
(Constant)	2.07	.31		6.58	.000
Screening location (HG=1; IG=0)	1.35	.500	.240	2.70	.008*
DOI	.001	.004	.028	.312	.755

NOTE. Dependent variable: nonsomatic symptom.

⁎ *P* value significant at <.05.

## Results

### Sample characteristics

Demographic and injury characteristics of study participants are summarized in table 1. The median age (interquartile range) across the group was 46 (28.3-66.3) years. Most individuals were men (71%), with paraplegia (53%), incomplete injuries (74%), and traumatic etiologies (59%). The HG and IG showed no significant differences in age, sex-at-birth, and injury characteristics, including rehabilitation LOS (*P*>.263). As expected, the DOI was significantly longer for the HG, as this included both time spent in rehabilitation and time spent at home postdischarge (*P*<.001; table 1).

### Comparisons of PHQ-9 scores and depressive symptomatology across groups

As can be seen from table 2, the rate of PMDD (PHQ-9 total score≥10) was higher in the HG (38%) versus IG (25.5%); although the difference was nonsignificant (χ^2^(1)=2.33; *P*=.09). Similarly, higher rates for more severe depression (moderate, moderately severe, and severe) and lower rates for no/minimal/mild depression were noted for the HG compared with the IG. An omnibus test, however, indicated nonsignificant differences across the groups (χ^2^(5)=3.72; *P*=.59). Consistent with these findings, the Mann-Whitney *U* test, which is based on ranked data also indicated statistically nonsignificant group differences between the HG (7.5 [4-12]) and IG (6.0 [3-10]) (*U=*1831; *P*=.09; table 2).

Comparison of subscale scores revealed significantly greater nonsomatic symptoms among individuals in the HG versus IG (*U=*1558; *P*=.004). There were no differences in the somatic subscale of the PHQ-9 (*U=*2125; *P*=.667; table 2). SI endorsement in the HG (21%) was noted to be higher than the 9% in IG (χ^2^(1)=3.87; *P*=.049). Comparison of median PHQ-9 (item 9) scores among those endorsing SI (N=19), noted the frequency/severity of SI in the HG to be significantly greater compared with the IG (*U*=24; *P*=.035).

Figure 2 describes post hoc exploratory analysis of PHQ-9 item endorsements across locations and their contribution to composite nonsomatic and somatic/cognitive symptoms. Only anhedonia differed significantly after Bonferroni correction. Other affective symptoms (depressed mood, worthlessness, and SI) were independently significant without correction (with *P*=.012, .049, and .049, respectively). All somatic/cognitive symptoms remained nonsignificant between the groups without the Bonferroni correction (*P*>.10).

Because DOI was significantly different between the groups, post hoc regression analyses to understand the influence of screening location on SI and nonsomatic symptoms, after adjusting for DOI, are presented in Table 3, Table 4. Screening location remained a significant predictor of nonsomatic symptoms (*P*=.008) as well as SI (*P*=.029) after DOI adjustment, with individuals in the HG on average showing 1.35 and 0.2 higher score points for nonsomatic symptoms and SI, respectively, compared with those in the IG.

## Discussion

This cross-sectional study compared psychological burden among individuals with SCI screened during inpatient rehabilitation (IG) and those screened postdischarge at home (HG) during the first year of injury, using a measure of depressive symptomatology (PHQ-9).

Investigation of the first study objective, comparing PHQ-9 total scores and their distribution across symptom severity categories, showed no significant differences between the HG and IG. A more nuanced exploration of symptom-specific analysis (somatic/cognitive vs nonsomatic), however, revealed marked differences in nonsomatic symptom scores that may have been otherwise masked by an aggregate single total PHQ-9 score. This aligns with previous research suggesting a cautious interpretation of a unitary PHQ-9 total score after SCI, as a single-factor model clustering all 9 items together was a poorer fit than a 2-factor model distinguishing somatic and nonsomatic symptoms.^37^^,^^40^ In addition, Krause et al^38^ noted that nonsomatic symptoms during inpatient rehabilitation predicted future depressive symptomatology contrary to somatic symptoms. The somatic items of the PHQ-9 can closely overlap with the immediate physiological effects of SCI, leading to difficulties in estimations of depression prevalence.^22^^,^^38^, ^39^, ^40^, ^41^ These findings underscore the importance of tracking nonsomatic symptoms to better characterize depression burden in early SCI.

This distinction likely also explains the findings of the second objective, where PMDD rates based on PHQ-9 total scores in the HG (38%) and IG (25.5%) did not significantly differ between the 2 groups (*P*=.09), despite a 12.5-percentage-point difference in rates. Comparable findings between the hospital and 6-month postdischarge setting have been previously noted.^22^ Yet, the worsening of depressive symptomatology after hospital discharge, in our study, may be significant based on significantly elevated nonsomatic scores in the HG. High psychological stress associated with a new SCI noted during hospitalization can persist or even increase in postdischarge settings.^11^ The transition from structured inpatient care to community reintegration can be a particularly vulnerable period after a SCI, marked by reduced support and continuity of care.^19^^,^^42^ Exploring psychological burden in the early phases of community reintegration is important as it can shape both immediate and long-term adjustments post-SCI.^13^^,^^16^^,^^19^^,^^22^, ^23^, ^24^^,^^27^^,^^28^^,^^43^

Understanding SI and suicide attempts in the first year post-SCI has been deemed critical for suicide prevention during both acute and chronic periods.^2^^,^^9^^,^^10^^,^^15^^,^^44^, ^45^, ^46^, ^47^, ^48^ Our third objective, comparing SI endorsement between locations, showed noteworthy differences in the rates of SI (21% in HG vs 9% in IG; *P*=.049), aligning with the observed higher nonsomatic subscale score in the HG. There is limited literature directly comparing SI prevalence during rehabilitation and postdischarge. Kishi et al^44^ noted that 13.3% of patients with SCI (n=60) had endorsed acute-onset SI during inpatient rehabilitation, whereas an additional 7% of individuals exhibited delayed-onset SI, 3-6 months postdischarge. The combined SI prevalence of 20% within the first 6 months of injury in their study seems to corroborate the 21% rate within the HG in our study. Kishi et al,^44^ moreover, noted that the individuals who developed delayed-onset SI had fewer predisposing risk factors (eg, psychiatric history, alcohol misuse) than those with acute-onset SI and that poorer social functioning in community settings was strongly associated with delayed-onset SI. A 2-year longitudinal study similarly noted high depression among 25% of SCI participants within 1 year of postinjury, 50% of whom developed delayed-onset depression postdischarge. In addition, those with delayed depression appeared to note poorer adaptation and elevated threat appraisal, compared with individuals with depression persisting from hospitalization through postdischarge.^20^ Although individuals flagged for SI or depression risk during hospitalization should be monitored in community settings, those without similar flags may be at risk of slipping through the cracks while still developing depression and/or suicidal behaviors. Addressing screening gaps during this critical period is essential to prevent delays in early intervention and reduce long-term psychological burden.

Our study findings suggest greater psychological vulnerability among newly injured SCI individuals discharging into their homes and communities. Despite the most specialized in-hospital rehabilitation care, newly injured individuals can remain unprepared physically and psychologically.^26^ The intensity and duration of psychosocial consequences can vary among patients and may last throughout their lives.^49^ If mental health burden remains unaddressed in the first year, it may contribute to longer-term sustained depressive symptoms. As such, depression and SI rates are estimated to be significantly greater among individuals with SCI in the community compared with the general US population.^2^^,^^7^^,^^8^^,^^14^ Untreated depression can further lead to poorer rehabilitation outcomes, self-management, reduced treatment adherence, and increased health care visits, as well as negatively impact health outcomes and life satisfaction.^27^^,^^50^^,^^51^ Therefore, systematic universal screening is crucial for identifying depression and SI risk in the community phase of the first year postdischarge to ensure optimal mental health care.^6^^,^^10^^,^^15^^,^^38^^,^^52^^,^^53^ Furthermore, the trajectory of specific examination of nonsomatic/affective symptomatology from the onset of SCI during rehabilitation through the first year of community reintegration may effectively reveal mental health fluctuations in postdischarge settings.

### Study limitations

Several limitations should be noted in this study. First, although the PHQ-9 is a validated tool for evaluating depression, it is not a diagnostic tool and only assesses depressive symptom severity. In addition, because PHQ-9 assessments were administered via both in-person and telephone modalities, these differences may have introduced variability in responses. Second, the cross-sectional nature of our study limits understanding of whether HG individuals also endorsed SI and elevated severity of nonsomatic symptoms during inpatient rehabilitation and vice versa. Third, the secondary data in this study were limited to basic demographic and injury-related characteristics, lacking relevant social, clinical, and psychological data (eg, pain, medication, medical and psychiatric history, and social participation) that may influence depressive symptomatology.^10^^,^^28^ The exclusion of non-English speakers, single-site design, and the high nonparticipation in this study may have increased the risk of selection bias, limiting the generalizability of findings. Fourth, we acknowledge that the 2-factor classification of the nonsomatic and somatic items (which included difficulty concentrating) used in our study, based on the factor structure by Krause et al,^37^ may not be consistently adopted across studies. Nevertheless, the 4 affective items remaining elevated in the HG compared with the IG, irrespective of the classification remains robust. Finally, data collection overlapped with the COVID-19 pandemic, which may have introduced unprecedented psychological stressors impacting depression symptomatology.^54^ However, average HG SI rate in our study (21%) appears comparable with rates reported both during the COVID-19 pandemic (19.7%) and outside of the pandemic (19%).^9^^,^^55^ It is difficult to determine the extent to which the pandemic may have skewed results in both groups. However, it is important to recognize that unforeseen emergencies such as COVID-19 may worsen depression among individuals with SCI, emphasizing the need for ongoing routine screening to mitigate the risk of elevated depression after discharge.

## Conclusions

The elevated nonsomatic depressive symptoms and SI rates in the HG highlight the significant mental health challenges that individuals with a new SCI may face when transitioning from inpatient rehabilitation to home settings. Specific monitoring of nonsomatic symptoms may accurately capture mental health burden during this critical period. Early screening and psychological support in the first year postinjury are crucial for reducing long-term mental health burden and costs.^28^ Tracking individual trajectories of somatic and nonsomatic depressive symptoms during the early phase of community reintegration can inform the temporal dynamics of depression and guide in planning appropriate screening strategies. Future interventions should focus on strengthening robust mental health screening strategies and support systems for individuals with SCI in postdischarge settings.

## Supplier

a. SPSS, version 27.0; IBM.

## Disclosure

The investigators have no financial or nonfinancial disclosures to make in relation to this project.
